# Cell cycle regulation by the Wee1 Inhibitor PD0166285, Pyrido [2,3-d] pyimidine, in the B16 mouse melanoma cell line

**DOI:** 10.1186/1471-2407-6-292

**Published:** 2006-12-19

**Authors:** Osamu Hashimoto, Masako Shinkawa, Takuji Torimura, Toru Nakamura, Karuppaiyah Selvendiran, Masaharu Sakamoto, Hironori Koga, Takato Ueno, Michio Sata

**Affiliations:** 1 Liver Cancer Division, Research Center for innovatve cancer therapy and Center of the 21st century COE program for medical Science, Kurume University, Kurume, Japan; 2 The division of Gastroenterology, Internal Medicine, Kurume University of medicine, Kurume, Japan

## Abstract

**Background:**

Wee1 kinase plays a critical role in maintaining G2 arrest through its inhibitory phosphorylation of cdc2. In previous reports, a pyridopyrimidine molecule PD0166285 was identified to inhibit Wee1 activity at nanomolar concentrations. This G2 checkpoint abrogation by PD0166285 was demonstrated to kill cancer cells, there at a toxic highest dose of 0.5 μM in some cell lines for exposure periods of no longer than 6 hours. The deregulated cell cycle progression may have ultimately damaged the cancer cells. We herein report one of the mechanism by which PD0166285 leads to cell death in the B16 mouse melanoma cell line.

**Methods:**

Tumor cell proliferation was determined by counting cell numbers. Cell cycle distribution was determined by flow cytometry. Morphogenesis analysis such as microtubule stabilization, Wee1 distribution, and cyclin B location were observed by immunofluorescence confocal microscopy. An immunoblot analysis of cdc2-Tyr15, cyclin D, E, p16, 21, 27, and Rb. A real-time PCR of the mRNA of cyclin D were completed.

**Results:**

In our experiment, B16 cells also dramatically abrogated the G2 checkpoint and were found to arrest in the early G1 phase by treatment with 0.5 μM for 4 hours observed by flow cytometry. Cyclin D mRNA decreased within 4 hours observed by Real-time PCR. Rb was dephosphrylated for 24 hours. However, B16 cells did not undergo cell death after 0.5 μM treatment for 24 hours. Immnofluoscence microscopy showed that the cells become round and small in the morphogenesis. More interesting phenomena were that microtubule stabilization was blocked, and Wee1 distribution was restricted after treatment for 4 hours.

**Conclusion:**

We analyzed the effect of Wee1 inhibitor PD0166285 described first by Wang in the G2 transition in the B16 melanoma cell line. The inhibitor PD0166285 abrogated G2/M checkpoint inducing early cell division. Moreover, we found that the treatment of cells with the inhibitor is related to microtubule stabilization and decrease in cyclin D transcription. These effects together suggest that Wee1 inhibitor may thus be a potentially useful anti-cancer therapy.

## Background

The progression of the mammalian cell cycle is controlled by the sequential activation of a series of cell cycle-dependent kinases (CDKs) [1]. Dysfunction of these molecular checkpoints results in the proliferation of cancer cells. In this context, an abrupt shift of the cell to mitosis from the G2 phase has received increasing attention, as have elements of the G2 checkpoint, particularly Wee1 [2].

The activation of the mitosis-promoting kinase cdc2 is required for transition from the G2 to the G1 phase in all eukaryotic cells. Cdc2 is subject to multiple levels of regulation, including association with its major partner B-type cyclin, reversible phosphorylation, and intracellular compartmentalization. After association of cdc2 with cyclin B, activity of cdc2-cyclin B is repressed to a basal level until G2/M transition, when the G2/M checkpoints are complete [3,4]. Phosphorylation of cdc2 at Thr-14 and Tyr-15 is critical in the repression of cdc2-cyclin B. The protein kinase Wee1 [5,6] phosphorylates at Tyr-15, while another protein kinase membrane-associated cdc2 tyrosine- and threonine-specific cdc2 inhibitor (Myt1) phosphorylates both site [6,7]. Cdc25C, on the other hand, is a phosphatase that dephosphrylates cdc2 at Thr-14 and Tyr-15. As a result cyclin B-cdc2 is activated and the cell cycle progresses. Because the Thr-14 and Tyr-15 phosphorylations are crucial for function of the G2/M checkpoint [8], induction of G2 arrest may require activation of Wee1 and Myt1 in addition to inactivation of Cdc25C [9].

Human Wee1 is inactivated through phosphorylation and protein degradation during the M phase. This degradation of Wee1, carried out through ubiquitination by cdc34 [10] and the ubiquitin ligase complex (Skp1, CDC53/Cullin, F-box protein) [11], also is regulated by cdc2-cyclin B [12]. Typically, irradiation-induced DNA damage favors inactivation of Cdc25C as follows. The mechanism by which Cdc25C is inactivated involves phosphorylation at Ser-215 catalyzed by Chk1/Chk2, and a 14-3-3 exportion from nuclei. The upstream kinase that activates Chk1 is ATM, which can be activated by DNA damage. Such Cdc25C inactivation helps to maintain cell cycle arrest by Wee1. Another possibly relevant pathway involves the DNA damage response kinases, checkpoint kinase (Chk1) and serine/threonine-protein kinase (Cds1), which directly phosphorylate Wee1. However, the physiologic significance of this phosphorylation remains obscure [13,14].

After mitosis, daughter cells adhere to the extracelluler matrix. Cyclin D, which acts to initiate the cell cycle, then is expressed. Cyclin D expression is important for progression through the G1 phase. Expressions of cyclin D increased due to various stimuli. Initially, cyclin D is increased by the Rac-integrin signal associated with cell-to-cell matrix adhesion. After some hours, cyclin D expression is regulated through Erk signaling by growth factors [15]. Cyclin D combines with CDK4/6, cyclin E/CDK2 and cyclin A/CDK2 [16]. The cell will then advance into the G1 phase. E2F, which regulates the trasnscription of various molecules, is activated through phosphorylation inactivating Rb by CDKs; cells then advance into the S phase [17].

PD0166285, a ppyrido [2,3-d] pyimidine compound, was developed [18] as an inhibitor of Wee1; inhibition is evident even at nanomolar concentrations. Irradiation induces DNA injury, so cell arrest which prevent cell apoptosis. This is one of the reasons that effect of inducing apoptosis to cancer cells is restricted in radiation therapy. In seven cancer cell lines, 0.5 μM of PD0166285 for short exposure period (2–4 hour) was found to dramatically inhibit the radiation-induced cdc2 phosphorylation, which otherwise would have resulted from Cdc25C inactivation. Before the G2/M transition leading to cell division, Wee1 reversibly arrests the cell cycle by inactivating cdc2 through phosphorylation at Tyr-15. PD0166285 may thus nullify the G2 checkpoint. Because Wee1 is inhibited, cancer cells do not undergo cell cycle arrest, so mitosis continues despite radiation-induced injury to DNA. However, in cells susceptible to p53 and the G1 check point killing, PD0166285 acts as a radiosensitizer promoting cell death. The sensitivity enhancement ratio was found to be 1.23 by a clonogenic assay. Interestingly, this radiosensitizing activity is p53 dependent, thus showing greater efficacy in cells where p53 is active. This inhibitor represents a novel class of anticancer drugs that selectively enhance cancer cell killing by conventional therapies.

However, PD0166285 kills tumor cells directly in some cell lines at a toxic highest dose of 0.5 μM for long exposure periods of no more than 6 hours with no relation of p53. These effects were observed in many cell lines such as ovarian carcinoma PA-1 cells, colon cancer HT29, HCT116, cervical cancer HeLa, lung carcinoma H460 [18], and hepatoma cell line Hep3B [19].

In this report, after treatment with PD0166285, B16 cells were also arrested in the early G1 phase within at least 4 hours. Here we indicate novel antiproliferative activities to block adhesion of the Wee1 inhibitor PD0166285 having shown microtubule destabilization, and depression of cyclin D expression.

## Methods

### Tumor cell line

B16 mouse melanoma cells were maintained in Dulbecco's modified Eagle's medium (DMEM); Life Technologies, (Grand Island, NY) supplemented with 10% fetal bovine serum, (FBS).

### Cell counting

Cell proliferation was analyzed by counting the cells. B16 cells (1 × 10^5 ^cells in 100 mm-dishes) were maintained in medium overnight. In addition, the cells were treated with (0, 0.5, 1.0, or 2.0 *μ*M) PD0166285 (including DMSO vehicle) for indicated times. The cells were washed twice with phosphate-buffered saline (PBS). Next, the cells were trypsinized, so the cell numbers in each dish were determined by using a computed cell-counter (Sysmex CDA-500) according to manufacturer's recommendation.

### Drug

The Wee1 inhibitor, PD0166285, was kindly provided by Pfizer (Ann Arbor, MI).

### Antibodies

The present study used: antibodies against cyclin D, cyclin, p16, Wee1, Rb, and anti-*α*-tubulin (Santa Cruz Biotechnology, Santa Cruz, CA), and against p21 and p27 (Transduction Laboratories, San Diego, CA). Phospho-Plus cdc2 (Tyr15) antibody kit was obtained from New England BioLab (Beverly, MA).

### Flow Cytometry and Cell Cycle Analysis

After treatment with 0.5 μM PD0166285, B16 cells were trypsinized, washed with PBS, and incubated in 0.2% Triton X-100 for 20 min at 37°C. After incubation, cells were treated with propidium iodideide (PI) and then subjected to DNA content analysis. PI florescence was analyzed with a FACScalibur flow Cytometer (Becton Dickinson). Findings characterizing flow for at least 20,000 cells were collected and analyzed with Cell Quest software (Becton Dickinson). Cell cycle distributions were calculated with Mod Fit LT software (Becton Dickinson).

### Morphologic observation by immunofluorescence confocal microscopy

Cells were incubated in glass-bottom microwell dishes (poly-d-Lysine coated, 35 mm) for at least 4 hours, with or without 0.5 μM PD166285. *α*-tubulin: Cells were washed with PBS, fixed in 4% paraformaldehyde in PBS for 20 min at room temperature, and permeabilized in 0.2% TritonX-100 for 10 min at room temperature. After treatment against nonspecific binding with blocking protein (DAKO, Carpenteria, CA) for 20 min at room temperature, cells were incubated with anti-*α*-tubulin antibody (1:400) overnight at 4°C and then with FITC-conjugated secondary antibody for 1 hour at room temperature. After washing with PBS, cells were incubated with PI for 5 min at room temperature to stain the nucleus. Observation was carried out with a laser-scanning confocal immunofluorescence microscope (Olympus, Tokyo, Japan). Wee1: Cells grown in glass bottom microwell dishes (poly-d-Lysine coated, 35 mm) were fixed in 50:50 (vol/vol) methanol/acetone and incubated with anti-Wee1 antibody at a 1:250 dilution overnight at 4°C. FITC-conjugated secondary antibody was then added for 1 hour at room temperature. Cells were counter stained with PI or double-stained with anti-*α*-tubulin as observed above.

### Immunoblotting analysis

Cells were lysed in lysis buffer (50 mM HEPES at pH 7.5, 250 mM NaCl, 20 mM EDTA, and 0.1% Nonidet P-40) additionally containing 1 mM phenylmethylsulfonyl fluoride, a protein inhibitor cocktail (Sigma, St. Louis, MO), 10 nM NaF, and 1 mM Na_3_VO_4_. Lysates were incubated at 4°C for 15 min and then cleared of cell debris by centrifugation at 14,000 g for 15 min at 4°C. Supernatants were subjected to protein determinations using a DC protein assay kit (Bio-RAD Laboratories, Hercules, CA) according to the manufacturer's instructions. Total cell lysates were then added to an equal volume of 2 × sample loading buffer containing 2% sodium dodecyl sulfate (SDS) and boiled for 5 min. Total cell protein was then loaded onto SDS-polyacrylamide gels for electrophoresis. Proteins were electrically transferred to Fluorotrans membranes for immunoblotting. The membrane was blocked for 1 hour at room temperature with 5% nonfat dry milk and then incubated with primary antibody overnight at 4°C. The membrane was washed, and incubated with horseradish peroxidase-labeled secondary antibody for 1 hour at room temperature. Finally the membrane was incubated with a detection reagent kit including luminol in an alkaline buffer, according to manufacturer's recommendations. (Amersham Pharmacia Biotech, Piscataway, NJ). Specific bands were visualized by allowing the membrane to expose blue- light-sensitive autoradiographic films.

### Total RNA extraction, cDNA synthesis, and reverse transcription (RT)-polymerase chain reaction (PCR)

For total RNA isolation, cultured cells were extracted using the Isogen method (Nippon Gene, Tokyo, Japan). RNA was quantified by spectrophotometry. Complementary DNA (cDNA) was synthesized using 2 μg of total RNA with random primers and Superscript III reverse transcriptase (Invitrogen, Carlsbad, CA). The RNA and primers were denatured by heating at 70°C for 10 min. The RT reaction mixture was then incubated for 30 min at 50°C, followed by 15 min at 70°C. A template-free control was processed for each experiment, establishing an absence of genomic contamination in the samples. The resulting cDNA then was amplified by PCR (20 cycles) with primer pairs specific for cyclin D, or glyceraldehyde-3-phosphate dehydrogenase. PCR products were resolved in 1.5% agarose gels and visualized by ethidium bromide staining with ultraviolet transillumination. PCR cycle conditions were 94°C for 30 s, 6°C for 30 s and 72°C for 1 min. The primer pair sequences were as follows: cyclin D, 5'-TCCGGAGACCGGCAGTACAG-3' and 5'-TTGCAGCAGCTCCTCGGGC-3' (512 bp), and GAPDH, 5'-ACCACAGTCCATGCCATCAC-3' and 5'-TCCACACCCTGTTGCTGTA-3' (452 bp).

### SYBR Green real-time quantitative PCR

For quantitative SYBR Green real-time PCR, 0.6 μl of the RT products were used for each reaction, carried out using a SYBR Green PCR Core Reagent kit (PE Biosystems, Warrington, UK) according to the protocol provided by the manufacturer. Optimal conditions were established for each specific gene. Primer sequences were designed for cyclin D as follows using Primer Express Software (PE Applied Biosystems, Foster City, CA): 5'-GCAGCACCCGGTCGTTGAGGA-3' and 5'-TCCGGAGACCGGCAGTACA-3' (180 bp). After quantitative PCR was performed, products were analyzed using the ABI PRISM 7000 Sequence Detection System (PE Applied Biosystems). A three-stage PCR cycle program provided by the manufacturer was used, as follows: 2 min at 50°C, 10 min at 95°C, and then 40 cycles of 15 s at 95°C and 1 min at 60°C. Reaction products were visualized on ethidium bromide-stained 1.5% agarose gels. Appearance of a single band of the correct molecular size confirmed PCR specificity. Results are representative of three independent experiments [20].

## Results

### PD0166285 suppressed cell proliferation by inducing G0/1 cell cycle arrest

**Figure 1:**
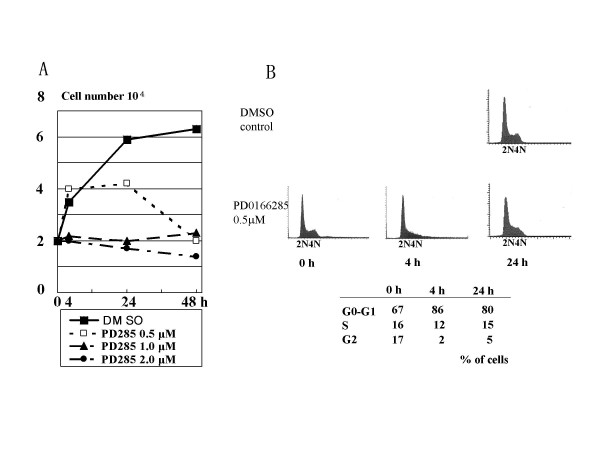
Effects of PD0166285 treatment on the suppression of cell proliferation and the cell cycle distribution. A: The cells were harvested after treatment with (0, 0.5, 1.0, or 2.0 μM) PD0166285 (including DMSO vehicle) for the indicated times, and then the cells were counted. B: The cells were harvested after treatment with 0.5 μM for the indicated period, fixed, stained with PI, and analyzed for DNA content by flow cytometry. The percent of cells in the cell cycle after treatment were indicated on lower part of the flow cytometry graphs.

Cell proliferation was analyzed by counting the cell numbers. We counted the cell number with DMSO control, 0.5, 1, and 2 μM treatment for 4 hours and 24 hours. 0.5 μM of PD0166285 was bottom line to anti-proliferate (Figure 1A). Cell cycle changes were investigated using flow cytometry. B16 cells were arrested in the early G1 stage after treatment with 0.5 μM PD0166285. No cells showing characteristics of apoptosis were observed (Figure 1B). The percentage of cells in the G0/G1 phase was 67% at 0 hours after PD0166285 treatment, and 86% at 4 hours. The percentage in the S phase was 16% at 0 hours, and 12% at 4 hours. The percentage in the G2 phase was 17% at 0 hours, and 2% at 4 hours. S-, G2-, and M-phase cells progressed to the early G1 phase within 4 hours. PD0166285 thus promoted complex cell cycle progression from G2 to the G1 phase in B16 cells. At 24 hours, the G1, S and G2 cell distributions were 81, 15 and 4%, respectively after PD0166285 treatment with 0.5 μM. In DMSO control, the percentage of cells was almost same within 0 h to 24 h. Sub-G1 which means that apoptosis was absent at 24 hours.

### PD0166285 inhibited microtubule stabilization

**Figure 2:**
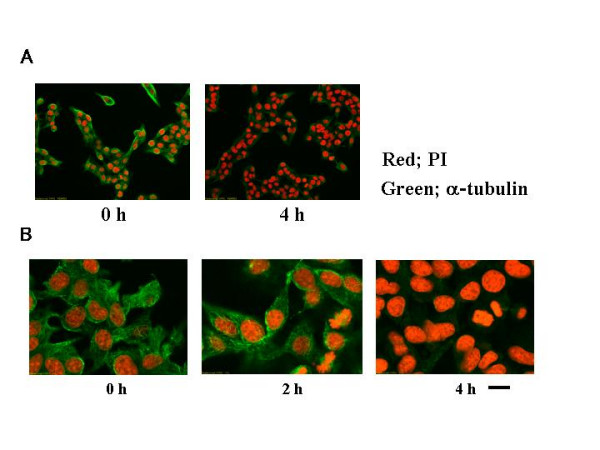
Effects of PD0166285 treatment on the microtubule stabilization. A: Cells were fixed and stained with PI (red) and anti-α-tubulin antibody followed by FITC-labeled secondary antibody (green). Shown is a typical image under laser confocal microscopy. B: magnifications at 0 h, 2 h, and 4 h. Bar: 10 μm

Double-staining immunofluorescence microscopy with PI (red) and anti-*α*-tubulin (green) after 4 hours of PD0166285 treatment is shown in Figure 2A. Nuclei in most of the cells had become small and the cell number had increased. However, the cells did not spread. Typical interphase microtubule networks had disappeared at 4 hours. Photomicrographs at 0 hours, 2 hours, and 4 hours show disappearances of microtubule networks in those cells not exhibiting mitosis (Figure 2B). PD0166285 thus inhibited microtubule stabilization. The amount of *α*-tubulin protein in cells with and without PD0166285 treatment remained the same (DATA not shown).

### PD0166285 inhibited Wee1 activity and progressed from the G2 to G1 phase

**Figure 3:**
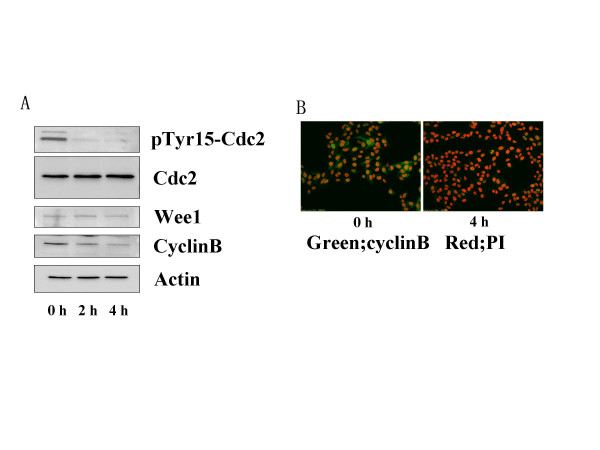
Effects of PD0166285 on the G2 phase. A: Immunoblot analysis of anti-cdc2, anti-phosphorylated cdc2-tyr15, and Wee1 was performed in lysates of with 0.5 μM PD0166285 treated for 0 h, 2 h, or 4 h. B: Subcellular location of cyclin B was observed. After 4 h of treatment with 0.5 μM PD0166285, cells were fixed and doubly stained for immunofluorescence with PI (red) and anti-cyclin B1 (green). Shown are typical merged images obtained by confocal microscopy. Bar: 10 μm.

PD0166285 almost completely abolished cdc2-Tyr15 phosporylation in B16 cells (Figure 3A). Expression of cdc2 were no change, but the expression of cyclin B decreased. We then characterized the cellular localization of cyclin B by laser confocal immunofluorescence microscopy. At 4 hours, cyclin B expression was diminished, although it could be seen in most cells in the early G1 phase (Figure 3B)

### Localization of Wee1 showed expression upon cell adhesion

**Figure 4:**
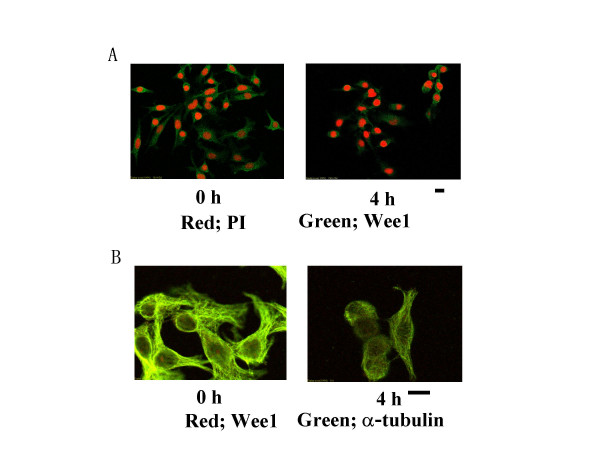
Effects of PD0166285 on the cellular location of Wee1 and adhesion. A: After 4 h of PD0166285 treatment, cells were fixed and doubly stained for immunofluorescence with PI (red) and anti-Wee1 (green). B: After 4 h of 0.5 μM PD0166285 treatment, morphologic changes and double stained with anti-α-tubulin (green) and anti-Wee1 (red) were observed. Shown are typical images obtained by laser confocal microscopy. Bar: 10 μm.

We characterized Wee1 in terms of its subcellular location in the cytoplasm, and also changes evident upon cell adhesion. Without PD0166285 treatment, Wee1 was seen extensively in the cytoplasm. Upon PD0166285 treatment, Wee1 was clustered tightly around nuclei while cells appeared shrunken (Figure 4A). Wee1 and *α*-tubulin were cellular co-localization molecules. After treatment, they were restricted in distribution. The spreading of cell and plasma membrane ruffling were suppressed in morphogenesis (Figure 4B). This indicated imperfect adhesion.

### Cell cycle-related proteins were altered

**Figure 5:**
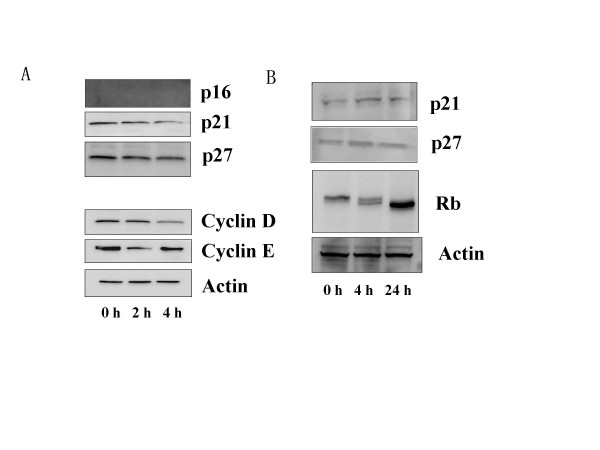
Effects of PD0166285 on expression related protein of cell cycle regulators. A: Lysates (30 μg) were prepared from cells after treatment with 0.5 μM PD0166285 for the indicated period (0 h, 2 h and, 4 h) were subjected to immunoblot analysis with anti-cyclin D, anti-cyclin E, anti-p16, anti-p21, and anti-p27 antibodies. B: Cell lysates after treatment with 0.5 μM PD0166285 for long periods treatment; (0 h, 4 h, and 24 h) were subjected to an immunoblot analysis with Anti-Rb antibody and cell cycle suppression directly molecule p21 and p27.

To address the mechanisms of cell cycle arrest induced by PD0166285 in B16 cells, expression of endogenous CDK inhibitors (CKIs) such as p16, p21, and p27 were investigated by immunoblot analysis with specific antibodies for CKIs (Figure 5A). P16 protein was not detected. P21 and p27 were detectable, but amounts were unchanged at also 24 houres (Figure 5B). Progression through the cell cycle is governed by kinase activities of specific CDKs, which are regulated by association with the regulatory cyclin subunits. We investigated cyclin D and E, which are related to the progression from the G1 to the S phase. The progression through the G1 phase involves mainly CDK4/6-cyclin D in the early G1 phase and CDK2-cyclinE in the mid G1 phase. Cyclin D decreased gradually until 4 hours. Cyclin E was found to decrease at 2 hours, but then it somewhat increased at 4 hours. P16, an inactivator of CDK4/6-cyclin D, was not detected in B16 cells. Therefore, it is known that cell cycle arrest is the suppression of cyclin D expression in B16 cells by PD0166285. We next investigated Rb, which regulates cell cycle progression from G1 to the S phase via E2F after treatment for 0, 4 and 24 hours. An immunoblot analysis showed a gradually inactive dephosphorylation of Rb, (Figure 5B).

### PD0166285 repressed cyclin D transcription

**Figure 6:**
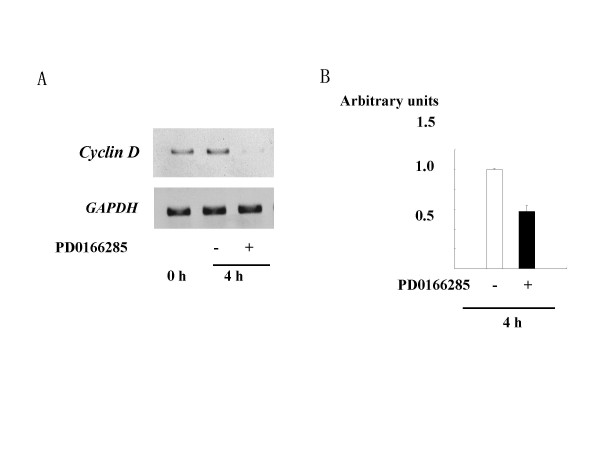
Effects of PD0166285 on cyclin D transcription. A: RT-PCR analysis of cyclin D mRNA in 0.5 μM PD0166285 treated cells. RT yielded a cDNA template for PCR amplification with specific-specific primers. GAPDH expression was analyzed as a control. B: Quantitative real-time PCR analysis of specific mRNA after treatment with 0.5 μM PD0166285. Three independent experiments were carried out.

We next investigated cyclin D mRNA transcription, using RT-PCR. Cyclin D mRNA transcription decreased after 4 hours of treatment with PD0166285 (Figure 6A). We then examined the transcription of cyclin D mRNA more quantitatively by real-time PCR. Expression of cyclin D mRNA decreased to 42.1% of baseline levels at 4 hours (Figure 6B). Thus, PD0166285 down-regulated the transcription of cyclin D mRNA within 4 hours, a time course similar to that of microtubule destabilization.

## Discussion

We studied the mechanisms by which the Wee1 inhibitor PD0166285 suppressed growth of the B16 mouse melanoma cell line. In previous reports, G2 checkpoint abrogation by 0.5 μM of PD0166285 was demonstrated to kill cancer cells [18,21]. Deregulated cell cycle progression ultimately may have damaged the cancer cells. When those authors examined toxicity, the highest dose of PD0166285 was 0.5 μM for exposure periods no more than 6 hours in some cell lines. When we exposed the hepatoma carcinoma cell line Hep3B to PD0166285, after 4 hours of treatment the Hep3B cells entered G1 arrest and after 6 hours the cells floated in the medium [19]. B16 melanoma cell line cells entered G1 arrested after 4 hours and the cells floated after 24 hours at 0.5 μM PD0166285. B16 cells showed an arrested cell proliferation for 4 hours, but no apoptosis was not observed after 24 hours of treatment by 0.5 μM PD0166285. After 24 hours, the cell were observed to be floating and dead (Figure 1A).

PD0166285 is the safe reagent. At least, 0.3 μM UCN-1 and 3 mM caffeine can induce the dephosphorylation of cdc2-Ty15. PD0166285 can induce the dephosphorylation at 0.5 μM which is very small concentrations. Moreover, for an in vivo assay, we injected 500 μl of 20 μM PD0166285 into the peritoneum cavity of B6 mice for 4 weeks. However the injected reagent was no negative effects to these mice (Data not shown). 0.5 μM PD0166285 might be therefore reagent with the peculiarity and safe for normal cells.

We could not investigate a human melanoma cell line and other cell lines, but such cell arrest and cell death could be observed in some cell lines including a human hepatoma cell line in the previous papers[18,19]. 0.5 μM PD0166285 thus appears to induce cell arrest in many cancer cell lines.

We found that PD0166285 rapidly brought about progression to cell cycle arrest in early G1 after a cell division phase. At a concentration of 0.5 μM, PD0166285 inhibited cdc2 inaction by phosphorylation at Ty15 (Figure 3A). It is reported that kinase activity of Wee1 is regulated by self-phosphorylation. At 4 hours, expression of Wee1 showed little change. Cyclin B protein expression was not seen by immunofluorescence microscopy (Figure 3B). B16 cells arrested in the early G1 phase at 4 hours, according to flow cytmetry (Figure 1). But cell death was not seen until 4 hours of exposure to PD0166285 at 0.5 μM.

These findings indicate that the effect of 0.5 μM PD0166285 for G1 cell arrest is Wee1 activity in an unusual way. The Wee1 knock down (siRNA experiment) might thus help to elucidate the direct or non-specific response. However, further investigation is called for.

The most surprising observation was made after 4 hours of treatment with 0.5 μM PD0166285. Observed by immunofluorescence microscopy, microtubule polymerization failed to occur in B16 cells treated with PD0166285. To clarify these observations we carried out immunofluorescence microscopy (Figure 2B), and flow cytometry (data not shown) after 2 hours of exposure. The cells in resting phase showed no cell cycle progression, and no clearly evident abnormalities. However, microtubule polymerization was blocked in all cells after 4 hours of PD0166285 treatment. Cyclin D was suppressed at 4 hours. PD0166285 treatment for 4 hours suppressed cyclin D protein expression and transcription. These observations indicated that PD0166285 arrested the cell cycle in the early G1 phase, accompanied by inhibition of microtubule stabilization and suppression of cyclin D expression by imperfect adhesion.

Changes in cell architecture are required for progression through the cell cycle. The most dramatic of these is a complete recognition of the cytoskeleton triggered by proteins phosphorylated by cyclinB-cdc2 such as the kinesin-like motor proteins required for proper assembly of mitotic spindle [22,23], as well as the actin binding protein caldesmon and several regulators of small GTPases, Rho and CDC42, that mediate rearrangements of the actin cytoskeleton during mitosis [24-26]. Recent data have indicated that plasma membrane lipid rafts are involved in signal transduction events; raft formation is initiated by cell adhesion to the extracelluler matrix, which is mediated by transmembrane proteins called integrins [27-29]. Lipid rafts are directed by integrins to target Rho and Rac GTPases. Microtubule stabilization is regulated by Rho-mDia signaling and Rac-PAK signaling [30], which therefore occur downstream of integrin-mediated cell adhesion via lipid raft formation. Focal adhesion kinase (FAK) has been described as a critical mediator of integrin signaling, allowing FAK to influence a variety of processes, including the broadening of the cell attachment, migration, survival and cycle progression.

Cyclin D expression begins to become evident after cell adhesion. Two pathways separately act on cyclin D expression at the early G1 and middle G1 phase. Early in G1, integrin-Rac is important for cyclin D expression when cell cycling is initiated after cell adhesion. Rac was reported to stimulate the expression of CCND1 mRNA in both fibroblasts and epithelial cells [31]. NF-κB has been implicated as Rac effectors [32,33]. Although the relationship between reactive oxygen species and cyclin D1 has yet to be elucidated, human and rodent CCND1 promoters contain functional NF-κB binding sites. In the mid G1 phase, some hours after the early G1 phase, several studies have shown that sustained signaling from extracelluler signal regulated kinases (ERK) stimulates the production of CCND1 mRNA in mesenchymal cells. Integrin signaling, which had originally induced cyclin D expression in early G1, is suppressed in the mid G1 phase [34]. Instead, various growth factors act via ERK to up-regulate cyclin D expression in mid G1 [35].

PD0166285 inhibited microtubule polymerization while suppressing cyclin D expression. Finally, Rb demonstrated inactive by dephosphorylation (Figure 5B), and the cell cycle was completely arrested in early G1 [36]. Importantly, adhesion-related molecules such as integrin, Rho, Rac, and FAK are highly complex and involve considerable cross talk in signaling [37]. Rho, Rac, and FAK activity were not analyzed in this study, but will be investigated in the future.

Microtubule stabilization failure might involve Wee1 inactivation or a direct drug effect of PD0166285 on microtubules. The role of Wee1 is not completely understood. Our result suggested there was Wee1 colocalization with *α*-tubulin (Figure 4B), as has been suggested previously [38]. Wee1 might affect tubulin directly, while other reports have indicated a relationship between Wee1 and the Src homologue protein Crk, which is associated with FAK signaling [39,40]. Recently, a novel Wee1 inhibitor related to compounds showing Src kinase homology was reported to arrest the cell cycle in the S phase[41]. Wee1 might act upon FAK signaling with respect to signal transmission involving a Src-homology protein. As a result, Wee1 may be more than a G2 checkpoint nullifier, since it also affects cell adhesion molecules.

## Conclusion

In this study we demonstrated a novel cell cycle-regulating effect of the Wee1 inhibitor PD0166285, a pyrido (2,3-d) pyimidine, in B16 mouse melanoma cells. PD0166285 inhibited Wee1 function, abrogated the G2 checkpoint, and induced early cell division. PD0166285 blocked microtubule stabilization, and inhibited cyclin D expression. These effects thus suggest that Wee1 inhibitor may thus be a potentially effective anti-cancer drug.

## Competing interests

The author(s) declare that they have no competing interests.

## Authors' contributions

O.H. designed and supervised the study and prepared the manuscript. M.S Performed cell culture and molecular experiments include western blot, Microscopy, etc. T.N performed real-time PCR. K.S assisted western blot. M.S, H.K, and T.T advised and assisted molecular experiments and analysis of cellular change by microscopy. T.U and M.S participated in design of the study. All authors read and approved the final manuscript.

## Pre-publication history

The pre-publication history for this paper can be accessed here:



## References

[B1] Sherr CJ (1996). Cancer cell cycles. Science.

[B2] Kawabe T ( 2004). G2 checkpoint abrogators as anticancer drugs. Mol Cancer Ther.

[B3] Nurse P (1990). Universal control mechanism regulating onset of M-phase. Nature.

[B4] Nurse P (1997). Checkpoint pathways come of age.. Cell.

[B5] Watanabe N, Broome M, Hunter T (1995). Regulation of the human WEE1Hu CDK tyrosine 15-kinase during the cell cycle. EMBO J.

[B6] Poon RY, Chau MS, Yamashita K, Hunter T (1997). The role of Cdc2 feedback loop control in the DNA damage checkpoint in mammalian cells.. Cancer Res.

[B7] Liu F, Rothblum-oviatt C, Ryan CE, Piwnica-Worms H (1999). Overproduction of Human Myt1 Kinase Induces a G2 Cell Cycle Delay by Interfering with the Intracellular Trafficking of Cdc2-Cyclin B1 Complexes. Mol Cell Biol.

[B8] Russell P (1998). Checkpoints on the road to mitosis.. Trends Biochem Sci.

[B9] Parker LL, Piwnica-Worms H (1992). Inactivation of the p34cdc2-cyclin B complex by the human WEE1 tyrosine kinase.. Science.

[B10] McGowan CH, Russell P (1995 ). Cell cycle regulation of human WEE1. EMBO J.

[B11] Michael WM, Newport J (1998). Coupling of mitosis to the completion of S phase through Cdc34-mediated degradation of Wee1.. Science.

[B12] Sia RAL, Bardes ESG, Lew DJ (1998). Control of Swe1p degradation by the morphogenesis checkpoint. EMBO J.

[B13] Boddy MN (1998). Replication checkpoint enforced by kinases Cds1 and Chk1. Science.

[B14] J.O’Connell M, M.Raleigh J, M.Verkade H, Nurse P (1997). Chk1 is a wee1 kinase in the G2 DNA damage checkpoint inhibiting cdc2 by Y15 phosphorylation. EMBO J.

[B15] Welsh CF, Roovers K, Villanueva J, Liu Y, Schwartz MA, Assoian RK (2001). Timing of cyclin D1 expression within G1 phase is controlled by Rho.. Nat Cell Biol.

[B16] Keenan SM, Lents NH, Baldassare JJ (2004). Expression of Cyclin E Renders Cyclin D-CDK4 Dispensable for Inactivation of the Retinoblastoma Tumor Suppressor Protein, Activation of E2F, and G1-S Phase Progression. J Biol Chem.

[B17] Gonza'lez T, Seoane M, Caamano P, Vinuela J, Domi´nguez F, Zalvide J (2003). Inhibition of Cdk4 Activity Enhances Translation of p27kip1 in Quiescent Rb-negative Cells. J Biol Chem.

[B18] Wang Y, Li J, Booher RN, Kraker A, Lawrence T, Leopold WR, Sun Y (2001). Radiosensitization of p53 Mutant cells by PD0166285, a novel G(2) Checkpoint Abrogator. Cancer RES.

[B19] Hashimoto O, Ueno T, Kimura R, Ohtsubo M, Nakamura T, Koga H, Torimura T, Uchida S, Yamashita K, Sata M (2003). Inhibition of proteasome-dependent degradation of Wee1 in G2-arrested Hep3B cells by TGF beta 1.. Mol Carcinog.

[B20] Nakamura T (2004). Suppression of transforming growth factor-beta results in upregulation of transcription of regeneration factors after chronic liver injury. J Hepatol.

[B21] Li J, Wang Y, Sun Y, Lawrence TS (2002). Wild-type TP53 inhibits G(2)-phase checkpoint abrogation and radiosensitization induced by PD0166285, a WEE1 kinase inhibitor.. Radiat Res.

[B22] Blangy A (1995). Phosphorylation by p34cdc2 regulates spindle association of human Eg5, a kinesin-related motor essential for bipolar spindle formation in vivo.. Cell.

[B23] Ubersax JA (2003). Targets of the cyclin-dependent kinase Cdk1.. Nature.

[B24] Ridley AJ, Schwartz MA, Burridge K, Firtel RA, Ginsberg MH, Borisy G, Parsons JT, Horwitz AR (2003). Cell migration: integrating signals from front to back.. Science.

[B25] Burridge K, Wennerberg K (2004). Rho and Rac take center stage. Cell.

[B26] Etienne-Manneville S, Hall A (2002). Rho GTPases in cell biology.. Nature.

[B27] del Pozo MA, Alderson NB, Kiosses WB, Chiang HH, Anderson RG, Schwartz MA (2004). Integrins regulate Rac targeting by internalization of membrane domains. Science.

[B28] Palazzo AF, Eng CH, Schlaepfer DD, Marcantonio EE, Gundersen GG (2004). Localized stabilization of microtubules by integrin- and FAK-facilitated Rho signaling.. Science.

[B29] del Pozo MA (2004). Integrin Signaling and lipid Rafts. Cell Cycle.

[B30] Guan J (2004). Cell biology. Integrins, rafts, Rac, and Rho. Science.

[B31] Roovers K, Assoian RK (2003). Effects of Rho Kinase and Actin Stress Fibers on Sustained Extracellular Signal-Regulated Kinase Activity and Activation of G1 Phase Cyclin-Dependent Kinases. Mol Cell Biol.

[B32] Kumar A, Murphy R, Robinson P, Wei L, Boriek AM (2004). Cyclic mechanical strain inhibits skeletal myogenesis through activation of focal adhesion kinase, Rac-1 GTPase, and NF-kappaB transcription factor.. FASEB J.

[B33] Murga C, Gutkind JS (2002). Rac1 and RhoG promote cell survival by the activation of PI3K and Akt, independently of their ability to stimulate JNK and NF-kappaB.. Oncogene.

[B34] Roovers K, Klein EA, Castagnino P, Assoian RK (2003). Nuclear translocation of LIM kinase mediates Rho-Rho kinase regulation of cyclin D1 expression.. Dev Cell.

[B35] Page K, Li J, Hodge JA, Liu PT, Hoek TLV, Becker LB, Pestel RG, Rosneri MR, Hershenson MB (1999). Characterization of a Rac1 Signaling Pathway to Cyclin D1 Expression in Airway Smooth Muscle Cells. J Biol Chem.

[B36] Connell-Crowley L, Harper JW, Goodrich"t DW (1997). Cyclin Dl/Cdk4 Regulates Retinoblastoma Proteinmediated Cell Cycle Arrest by Site-specific Phosphorylation. Mol Biol Cell.

[B37] Schwartz MA, Ginsberg MH (2002). Networks and crosstalk: integrin signalling spreads.. Nat Cell Biol.

[B38] Baldin V, Ducommun B (1995). Subcellular localisation of human wee1 kinase is regulated during the cell cycle. J Cell Science.

[B39] Smith JJ, Evans EK, Murakami M, Moyer MB, Kornbluth S, M. Arthur Moseley, George Vande Woude (2000 ). Wee1-regulated Apoptosis Mediated by the Crk Adaptor Protein in Xenopus Egg Extracts. J Cell Biol.

[B40] Smith JJ, Richardson DA, Kopf J, Hollingsworth RE, Kornbluth S, Minoru Yoshida (2002). Apoptotic Regulation by the Crk Adapter Protein Mediated by Interactions with Wee1 and Crm1/Exportin. Mol Cell Biol.

[B41] Mizenina OA, Moasser MM (2004). S-phase Inhibition of Cell Cycle Progression by a  Novel Class of Pyridopyrimidine Tyrosine Kinase Inhibitors. Cell Cycle.

